# The complete mitochondrial genome of *Apis mellifera unicolor* (Insecta: Hymenoptera: Apidae), the Malagasy honey bee

**DOI:** 10.1080/23802359.2019.1671247

**Published:** 2019-09-30

**Authors:** Leigh Boardman, Amin Eimanifar, Rebecca T. Kimball, Edward L. Braun, Stefan Fuchs, Bernd Grünewald, James D. Ellis

**Affiliations:** a Honey Bee Research and Extension Laboratory, Entomology and Nematology Department, University of Florida, Gainesville, FL, USA;; b Independent Senior Research Scientist, Industrial District, Easton, MD, USA;; c Department of Biology, University of Florida, Gainesville, FL, USA;; d Institut für Bienenkunde, Polytechnische Gesellschaft, Goethe-Universität Frankfurt am Main, Oberursel, Germany

**Keywords:** Mitogenome, next-generation sequencing, A-lineage honey bee

## Abstract

The complete mitochondrial genome of the endemic Malagasy honey bee *Apis mellifera unicolor* is 16,373 bp and comprises 13 protein-coding genes, 22 transfer RNA genes, two ribosomal RNA genes, and a control region. The mitochondrial genome closely resembles mitogenomes of other published *Apis mellifera* subspecies, and the phylogenetic analysis suggests that *A. m. unicolor* is distinct from other African (A) lineage honey bees but is most closely related to the honey bees from southern African: *A. m. scutellata* and *A. m. capensis*.

*Apis mellifera unicolor* (Latreille, 1804) is endemic to Madagascar, an island off the East Coast of Africa. This subspecies is most closely related to other African honey bees (Ruttner 1988; Han et al. 2012). Despite interest in this honey bee, and studies on specific mitochondrial genes (Franck et al. 2001; Rasolofoarivao et al. 2015; Techer et al. 2017), the complete annotated mitochondrial genome of *A. m. unicolor* is not available. Here, we sequenced the mitogenome (GenBank: MN119925) of a worker *A. m. unicolor* honey bee from the Ruttner Bee Collection at the Bee Research Institute at Oberursel, Germany (Voucher No. 2520, Ch. Delaine, 1998, Madagascar, 18°48S, 47°38E). Identification was confirmed morphometrically. Genomic DNA extraction and quantification, genomic library preparation, and next-generation Illumina Hi-Seq 3000/4000 (San Diego, CA) sequencing with PE150 were performed following Eimanifar et al. (2017).

Sequencing data were quality checked using FastQC (Andrews 2010) and trimmed with Trimmomatic (Bolger et al. 2014). The resulting reads were mapped individually to eight existing *A. mellifera* mitogenomes in Geneious Prime 2019.0.4 (Kearse et al. 2012) using medium-low sensitivity and up to five iterations. The consensus sequence from the highest pairwise identity – in this case, *A. m. lamarckii* (KY464958) – was used as the reference sequence for a second round of mapping. The assembled mitogenome was annotated in mitos2 (Bernt et al. 2013) and manually adjusted to the *A. m. capensis* (KX870183) annotation in Geneious Prime. The 13 protein-coding genes (PCGs) and two ribosomal RNA (rRNA) genes were manually aligned with existing *Apis* mitogenomes in Mesquite v3.5 (Maddison and Maddison 2018). The phylogenetic estimation used RAxML 8.2.10 (Stamatakis 2014) with the GTRGAMMA model and 1000 bootstrap replicates (-f a option) and was run on CIPRES Science Gateway V. 3.3 (Miller et al. 2010). *P*-distances were generated using PAUP 4.0a (Swofford 2003).

The complete mitogenome of the Malagasy honey bee *A. m. unicolor* is 16,373 bp, with an overall base composition of 43.3% A, 41.3% T, 9.7% C, and 5.6% G. The mitogenome consists of 13 PCGs, 22 transfer RNA (tRNA) genes, two rRNA genes, and one putative control region (CR). Nine PCGs are encoded on the light strand, and the remaining four PCGs (*nad1*, *nad4*, *nad4l*, and *nad5*) and two rRNAs are on the heavy strand. Two PCGs overlap, with *atp8* and *atp6* sharing 19 nucleotides. Six PCGs start with ATT, while four start with ATG, two used ATA, and one used ATC. All PCGs ended with a TAA stop codon. The 12S rRNA was 785 bp (81.0% AT), and the 16S rRNA was 1330 bp (83.9% AT). The 22 tRNAs identified ranged in size from 62 bp (tRNA-Gln) to 78 bp (tRNA-Thr), and all folded into typical cloverleaf structures.

Phylogenetic comparison of the *A. m. unicolor* mitogenome showed that it is somewhat isolated from the other African honey bees (Figure 1). The taxa with the lowest *P*-distances from *A. m. unicolor* were *A. m. scutellata* (0.0074) and *A. m. capensis* (0.0075). Sequencing additional *A. mellifera* subspecies mitogenomes will increase our understanding of mitochondrial diversity and evolution of *A. mellifera*.

**Figure 1.**
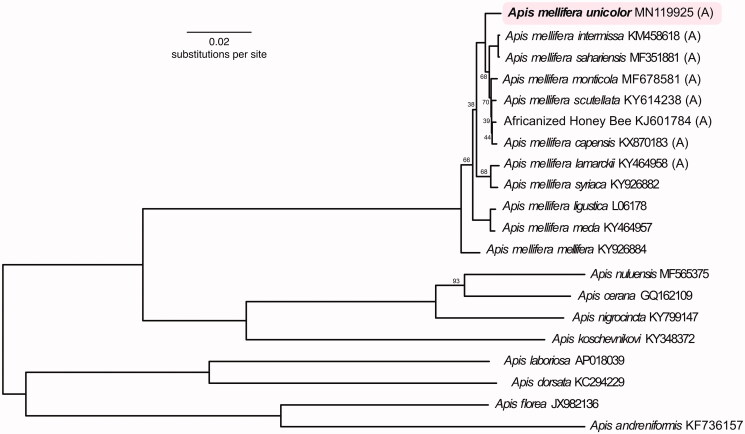
Phylogenetic tree showing the relationship between *Apis mellifera unicolor* (GenBank: MN119925) and 19 other *Apis* honey bee mitochondrial genomes (GenBank accession numbers are listed after species names). (A) indicates honey bees from the African A-lineage. The tree is midpoint rooted. Node labels indicate bootstrap values, and unlabeled lineages are 100%.
